# Adipose tissue lactate clearance but not blood lactate clearance is associated with clinical outcome in severe sepsis or septic shock during the post-resuscitation period

**DOI:** 10.1186/cc13362

**Published:** 2014-03-17

**Authors:** M Theodorakopoulou, S Orfanos, N Nikitas, D Vasilliadi, S Apolonatou, A Diamantakis, G Karkouli, C Diakaki, A Armaganidis, I Dimopoulou

**Affiliations:** 1 University Hospital of Athens Greece 'ATTIKON', Attiki, Greece

## Introduction

Blood lactate clearance, a surrogate of tissue hypoxia, is associated with increased mortality in septic patients. However, no study has directly measured lactate clearance at the tissue level in the post-resuscitation period of sepsis. This study aimed to examine the relative kinetics of blood and tissue lactate clearances and to investigate whether these are associated with outcome in ICU patients having severe sepsis or septic shock during the post-resuscitation phase.

## Methods

A microdialysis catheter was inserted in the subcutaneous adipose tissue of the upper thigh and interstitial fluid samples were collected. Serial measurements of blood and interstitial fluid lactate levels were performed over a 48-hour period. Lactate clearance was calculated according to the formula: (lactate (initial) - lactate (delayed) / lactate (initial)) × 100%. Lactate (initial) is blood or tissue lactate within the first 24 hours after ICU admission (H0). Lactate (delayed) is blood or tissue lactate at H4, H8, H12, H16, H20, H24 and H48 (H = hours).

## Results

A total of 112 patients having septic shock (*n *= 79) or severe sepsis (*n *= 33) were examined. Tissue lactate clearance was higher compared with blood lactate clearance at H0 to H8 (*P *= 0.02), H0 to H12 (*P *= 008), H0 to H16 (*P *= 0.01), H0 to H20 (*P *= 0.01), and H0 to H24 (*P *= 0.02). Tissue lactate clearance was higher in survivors compared with nonsurvivors at H0 to H12, H0 to H20 and H0 to H24 (*P *= 0.02, for all). Multivariate analysis showed that ARACHE II along with tissue clearances at H0 to H12, H0 to H20 and H0 to H24 <30% were independent outcome predictors. Blood lactate clearance was not related to survival.

## Conclusion

In critically ill septic patients, after the initial resuscitation phase, adipose tissue clears lactate earlier than blood. High tissue lactate clearance, but not blood lactate clearance, is associated with a favorable clinical outcome.

